# Using machine learning to predict lymph node metastasis in patients with renal cell carcinoma: A population-based study

**DOI:** 10.3389/fpubh.2023.1104931

**Published:** 2023-03-24

**Authors:** Yuhan Zhang, Xinglin Yi, Zhe Tang, Pan Xie, Na Yin, Qiumiao Deng, Lin Zhu, Hu Luo, Kanfu Peng

**Affiliations:** ^1^Department of Nephrology, Third Military Medical University Southwest Hospital, Chongqing, China; ^2^Department of Respiratory Medicine Center, Third Military Medical University Southwest Hospital, Chongqing, China

**Keywords:** renal cell carcinoma, lymph node, metastasis, machine learning, online calculator

## Abstract

**Background:**

Lymph node (LN) metastasis is strongly associated with distant metastasis of renal cell carcinoma (RCC) and indicates an adverse prognosis. Accurate LN-status prediction is essential for individualized treatment of patients with RCC and to help physicians make appropriate surgical decisions. Thus, a prediction model to assess the hazard index of LN metastasis in patients with RCC is needed.

**Methods:**

Partial data were extracted from the Surveillance, Epidemiology, and End Results (SEER) database. Data of 492 individuals with RCC, collected from the Southwest Hospital in Chongqing, China, were used for external validation. Eight indicators of risk of LN metastasis were screened out. Six machine learning (ML) classifiers were established and tuned, focused on predicting LN metastasis in patients with RCC. The models were integrated with big data analytics and ML algorithms. Based on the optimal model, we developed an online risk calculator and plotted overall survival using Kaplan–Meier analysis.

**Results:**

The extreme gradient-boosting (XGB) model was superior to the other models in both internal and external trials. The area under the curve, accuracy, sensitivity, and specificity were 0.930, 0.857, 0.856, and 0.873, respectively, in the internal test and 0.958, 0.935, 0.769, and 0.944, respectively, in the external test. These parameters show that XGB has an excellent ability for clinical application. The survival analysis showed that patients with predicted N1 tumors had significantly shorter survival (*p* < 0.0001).

**Conclusion:**

Our study shows that integrating ML algorithms and clinical data can effectively predict LN metastasis in patients with confirmed RCC. Subsequently, a freely available online calculator (https://xinglinyi.shinyapps.io/20221004-app/) was built, based on the XGB model.

## 1. Introduction

Renal cell carcinoma (RCC) roughly comprises 90% of kidney cancer and the incidence of RCC is currently increasing (1). According to a recent report, there were nearly 400,000 new cases and 170,000 kidney cancer-related deaths worldwide in 2018 (2). Clear-cell RCC is the main type of RCC; other subtypes include papillary RCC and chromophobe RCC (3).

Although, with the gradual improvement in imaging detection, the clinical diagnosis and treatment rate of RCC are increasing annually, metastatic RCC is associated with poor prognosis (4, 5). Numerous previous studies have shown that in RCC, lymph node (LN) metastasis is strongly associated with distant metastasis. Clinically, accurate LN status assessment can improve the diagnosis and treatment of RCC (6, 7). Babaian et al. (8) noted that LN dissection (LND) can improve the 5-year survival rate of patients with LN involvement, suggesting that patients with renal cancer undergoing LND may have better survival. LN infiltration is one of the most important predictors of tumor progression and mortality (9). Therefore, it is imperative to investigate the strong predictors of LN metastasis and accurately identify at-risk patients who require LND.

Unclear imaging findings and low intraoperative positive biopsy rates have delayed the diagnosis and identification of early LN metastasis in RCC, thus limiting the therapeutic effect of LND (10). Although computed tomography (CT) and magnetic resonance imaging (MRI) can detect LNs ~1 cm in diameter, enlarged LNs are not necessarily a sign of metastasis. The specificity of using only a single imaging method to diagnose LN metastasis is poor. Published literature suggests that use of a combination of MRI and F-fluoro-2-deoxyglucose positron emission tomography (FDG-PET) may provide the highest accuracy, however, the capacity to perform such imaging is limited by the high cost and the limited availability of the required equipment (6). Therefore, a reliable and accurate predictive tool for the screening of high-risk populations and risk evaluation of LN metastasis in RCC is urgently required. To date, few studies have assessed the predictors of LN metastasis in patients with RCC.

The Surveillance, Epidemiology, and End Results (SEER) database was developed by the US National Cancer Institute and is available for open access analysis. We aimed to build a reliable and accurate tool based on machine learning (ML) algorithms using an extensive number of patients with RCC from the SEER database and the Southwest Hospital in Chongqing, China, for use in screening patients at high risk of developing LN metastasis.

## 2. Materials and methods

### 2.1. Patient information

Patient information was obtained from the SEER research database, which is widely used for the analysis of clinical cancer databases worldwide. Information on LN status was obtained from records of SEER database variables based on the seventh edition of the American Joint Committee on Cancer (AJCC) tumor-node-metastasis (TNM) staging; The records had sufficient data on imaging and pathological findings to enable LN status to be assessed. The inclusion criterion was patients in the SEER database with histologically diagnosed kidney cancer diagnosed from 2010 to 2017. Patients with any of the following exclusion criteria were excluded: (1) age younger than 20 years, (2) unknown tumor laterality, (3) unknown tumor size, (4) unknown TNM stage, (5) more than one primary tumor, or (6) unknown tumor grade.

**Figure 1:**
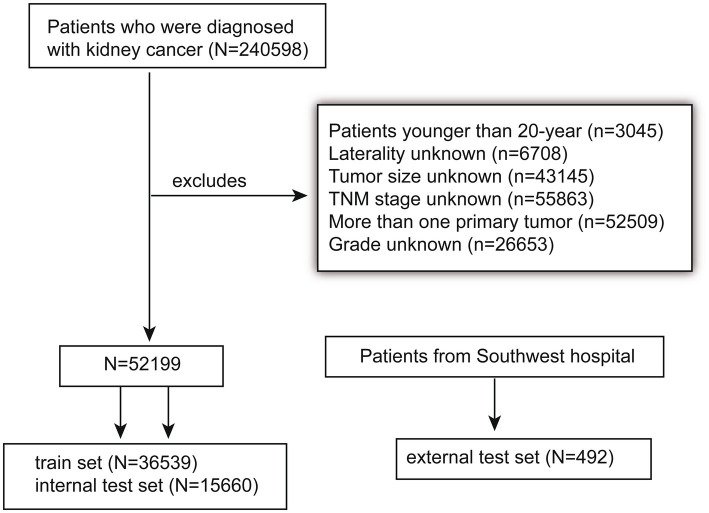
Patient selection flowchart.

As a result, 52,199 eligible patients were enrolled in this retrospective cohort study. Subsequently, we randomly divided the data of these patients into a training set (*N* = 36,539) and an internal test set (*N* = 15,660). Based on the training cohort, we constructed a predictive model by combining clinicopathological variables and the 7th TNM classification of the AJCC. In addition, the data of 492 patients from the Southwest Hospital in Chongqing, China were utilized as the external validation cohort to further validate the applicability of ML models. The process of selecting data for the study is shown in Figure 1. Three investigators collated the data, of whom two were responsible for data extraction, and the other investigator performed accuracy checks.

### 2.2. Data preprocessing and feature selection

All common features, including age, sex, tumor laterality, TNM stage, tumor size, tumor histology, race, and tumor grade, included in the analysis were reviewed by clinicians and filtered using the SEERStat software (8.4.0.1 version). Age was classified in four categories: <50, 50–59, 60–69, and ≥70 years. TNM stage was classified according to the 7th AJCC TNM classification. Based on the World Health Organization classification scheme, histological categories included the following: 8120, transitional cell carcinoma; 8,255, adenocarcinoma with mixed subtypes; 8,260, papillary adenocarcinoma; 8,310, clear-cell adenocarcinoma; 8,312, RCC; and 8,317, chromophobe renal carcinoma and other rare subtypes. The chi-squared test was applied to evaluate the differences between categorical variables, and the *t*-test was applied to analyze the differences between continuous variables. Univariable logistic regression (LR) analysis was performed to evaluate the risk factors for predicting LN metastasis in the training cohort of patients with RCC. Statistical significance was set at *p* < 0.05. The odds ratio (OR) was calculated using backward stepwise selection with the 95% confidence interval (CI). All statistical analyses were performed using R software version 4.2.1 (R Foundation for Statistical Computing, Vienna, Austria).

### 2.3. Model development and performance assessments

ML algorithms, a form of artificial intelligence, generally transcend traditional regression methods in predicting outcomes. The following six ML algorithms were used to build the models: LR, extreme gradient-boosting (XGB), random forest, support vector machine, artificial neural network (ANN), and decision tree models. The area under the receiver operating characteristic (ROC) curve (AUC), accuracy, sensitivity, and specificity were included in the study to assess the predictive power of each ML algorithm. The decision clinical analysis (DCA) curve and clinical utility curve (CUC) were utilized to examine clinical applicability. Furthermore, based on the best-performing model, we built a web-based online calculator and used the Kaplan–Meier method to predict the overall survival (OS) and to compare the survival outcome between patients with predicted N1 and N0 tumors.

### 2.4. Correlation analysis and variable importance

After features were screened out using univariable LR, we performed a Spearman correlation analysis to evaluate the relevant degree among these variables. The relevant index included three levels: 0–0.4, low; 0.4–0.7, intermediate; and ≥0.7, high. The relative variable importance was ranked using the permutation method in the best-performing model.

## 3. Results

### 3.1. Clinical characteristics of patients

After screening, 52,691 patients were enrolled, and 10 variables were included in this study. There were no significant differences between the training and internal validation cohorts in any of these characteristics (Table 1). Owing to geographic and ethnic differences and the limited sample size, there were statistically significant differences between the external validation cohort and training set, in most variables except for sex and N stage. In addition, there were no statistically significant differences in variable distribution between patients with and without LN metastases (Table 2).

**Table 1:** Characteristics in the training, internal test, and external test cohorts.

	Training set (*N* = 36,539)	Internal test (*N* = 15,660)	Value of *p*	External test (*N* = 492)	Value of *p*
Age (years)			0.1853		0.002
<50	7,505 (20.5%)	3,192 (20.4%)		78 (15.9%)	
≥70	8,024 (22.0%)	3,451 (22.0%)		107 (21.7%)	
50–60	9,851 (27.0%)	4,350 (27.8%)		172 (35.0%)	
60–70	11,159 (30.5%)	4,667 (29.8%)		135 (27.4%)	
Sex			0.704		0.615
Female	13,580 (37.2%)	5,792 (37.0%)		176 (35.8%)	
Male	22,959 (62.8%)	9,868 (63.0%)		316 (64.2%)	
Tumor laterality			0.886		0.01
Left	17,984 (49.2%)	7,719 (49.3%)		272 (55.3%)	
Right	18,555 (50.8%)	7,941 (50.7%)		220 (44.7%)	
T stage			0.235		< 0.001
T1	24,400 (66.8%)	10,314 (65.9%)		385 (78.3%)	
T2	3,891 (10.6%)	1713 (10.9%)		42 (8.5%)	
T3	7,576 (20.7%)	3,330 (21.3%)		53 (10.8%)	
T4	672 (1.8%)	303 (1.9%)		12 (2.4%)	
Tumor size, mean (SD)	53.5 (37.7)	54.0 (36.8)	0.211	47.4 (23.2)	< 0.001
Tumor histology			0.303		< 0.001
8,120	132 (0.4%)	49 (0.3%)		0 (0%)	
8,255	916 (2.5%)	428 (2.7%)		0 (0%)	
8,260	4,190 (11.5%)	1,813 (11.6%)		27 (5.5%)	
8,310	24,794 (67.9%)	10,629 (67.9%)		442 (89.8%)	
8,312	3,983 (10.9%)	1,731 (11.1%)		23 (4.7%)	
8,317	1,588 (4.3%)	622 (4.0%)		0 (0%)	
Others	936 (2.6%)	388 (2.5%)		0 (0%)	
Tumor grade			0.222		< 0.001
I	4,149 (11.4%)	1,697 (10.8%)		90 (18.3%)	
II	18,561 (50.8%)	7,920 (50.6%)		238 (48.4%)	
III	10,696 (29.3%)	4,664 (29.8%)		127 (25.8%)	
IV	3,133 (8.6%)	1,379 (8.8%)		37 (7.5%)	
M stage			0.323		0.004
M0	33,590 (91.9%)	14,355 (91.7%)		469 (95.3%)	
M1	2,949 (8.1%)	1,305 (8.3%)		23 (4.7%)	
Race					
Asian	2,198 (6.0%)	935 (6.0%)	0.372	492 (100.0%)	< 0.001
Black	3,960 (10.8%)	1,738 (11.1%)		——	
Others	700 (1.9%)	331 (2.1%)		——	
White	29,681 (81.2%)	12,656 (80.8%)		——	
N stage			0.215		0.521
N0	34,961 (95.7%)	14,945 (95.4%)		466 (94.7%)	
N1	1,578 (4.3%)	715 (4.6%)		26 (5.3%)	

SD, standard deviation.

**Table 2:** Characteristics of the patients presenting with and without lymph node metastases.

Variables	Overall (*N* = 52,691)	N0 (*N* = 50,372)	N1 (*N* = 2,319)	Value of *p*
Age (years)				<0.001
<50	10,775 (20.4%)	10,386 (20.6%)	389 (16.8%)	
≥70	11,582 (22.0%)	11,036 (21.9%)	546 (23.5%)	
50–60	14,373 (27.3%)	13,745 (27.3%)	628 (27.1%)	
60–70	15,961 (30.3%)	15,205 (30.2%)	756 (32.6%)	
Sex				<0.001
Female	19,548 (37.1%)	18,846 (37.4%)	702 (30.3%)	
Male	33,143 (62.9%)	31,526 (62.6%)	1,617 (69.7%)	
Tumor laterality				<0.001
Left	25,975 (49.3%)	24,702 (49.0%)	1,273 (54.9%)	
Right	26,716 (50.7%)	25,670 (51.0%)	1,046 (45.1%)	
T stage				<0.001
T1	35,099 (66.6%)	34,855 (69.2%)	244 (10.5%)	
T2	5,646 (10.7%)	5,342 (10.6%)	304 (13.1%)	
T3	10,959 (20.8%)	9,585 (19.0%)	1,374 (59.2%)	
T4	987 (1.9%)	590 (1.2%)	397 (17.1%)	
Tumor size, mean (SD)	53.6 (37.3)	51.5 (35.3)	99.2 (49.3)	<0.001
Tumor histology				<0.001
8,120	181 (0.3%)	114 (0.2%)	67 (2.9%)	
8,255	1,344 (2.6%)	1,199 (2.4%)	145 (6.3%)	
8,260	6,030 (11.4%)	5,744 (11.4%)	286 (12.3%)	
8,310	35,865 (68.1%)	34,798 (69.1%)	1,067 (46.0%)	
8,312	5,737 (10.9%)	5,336 (10.6%)	401 (17.3%)	
8,317	2,210 (4.2%)	2,174 (4.3%)	36 (1.6%)	
Others	1,324 (2.5%)	1,007 (2.0%)	317 (13.7%)	
Tumor grade				<0.001
I	5,936 (11.3%)	5,893 (11.7%)	43 (1.9%)	
II	26,719 (50.7%)	26,403 (52.4%)	316 (13.6%)	
III	15,487 (29.4%)	14,497 (28.8%)	990 (42.7%)	
IV	4,549 (8.6%)	3,579 (7.1%)	970 (41.8%)	
Race				0.219
Asian	3,625 (6.9%)	3,464 (6.9%)	161 (6.9%)	
Black	5,698 (10.8%)	5,424 (10.8%)	274 (11.8%)	
Others	1,031 (2.0%)	995 (2.0%)	36 (1.6%)	
White	42,337 (80.3%)	40,489 (80.4%)	1,848 (79.7%)	
M stage				<0.001
M0	48,414 (91.9%)	47,489 (94.3%)	925 (39.9%)	
M1	4,277 (8.1%)	2,883 (5.7%)	1,394 (60.1%)	

SD, standard deviation.

### 3.2. Univariable and multivariable logistic regression analysis

Eight risk factors associated with LN metastases, including age, sex, tumor laterality, T stage, tumor size, tumor histology, tumor grade, and M stage, were identified using univariable LR analysis (Table 3). In this study, ML algorithms utilized these risk factors to develop six available models. In the multivariable LR analysis, age (50–59 years) and tumor laterality (right) were dependent protective factors for LN metastasis, and T stage (T2, T3, T4), M stage (M1), tumor grade (III, IV), and tumor histology of transitional cell carcinoma were independent risk factors for LN metastasis.

**Table 3:** Univariable and multivariable logistic regression analyses of risk factors for LN metastasis in patients with RCC.

Variables	Univariable		Multivariable	
	OR	Value of *p*	OR	Value of *p*
Age (years)				
<50	Reference	Reference	Reference	Reference
≥70	**1.337**	**< 0.0001**	0.867	0.144
50–60	**1.194**	**0.027**	**0.774**	**0.007**
60–70	**1.367**	**< 0.0001**	0.931	0.435
Sex				
Female	Reference	Reference	Reference	Reference
Male	**1.349**	**< 0.0001**	1.014	0.833
Tumor laterality				
Left	Reference	Reference	Reference	Reference
Right	**0.784**	**< 0.0001**	**0.83**	**0.002**
T stage				
T1	Reference	Reference	Reference	Reference
T2	**7.718**	**< 0.0001**	**2.813**	**< 0.0001**
T3	**20.339**	**< 0.0001**	**5.834**	**< 0.0001**
T4	**86.644**	**< 0.0001**	**7.889**	**< 0.0001**
Tumor histology				
8,120	Reference	Reference	Reference	Reference
8,255	**0.184**	**< 0.0001**	**0.264**	**< 0.0001**
8,260	**0.081**	**< 0.0001**	0.492	0.002
8,310	**0.051**	**< 0.0001**	**0.136**	**< 0.0001**
8,312	**0.123**	**< 0.0001**	**0.31**	**< 0.0001**
8,317	**0.027**	**< 0.0001**	**0.115**	**< 0.0001**
Others	**0.526**	**0.001**	0.473	0.001
Tumor size, mean (SD)	**1.023**	**< 0.0001**	**1.005**	**< 0.0001**
M stage				
M0	Reference	Reference	Reference	Reference
M1	**25.102**	**< 0.0001**	**7.515**	**< 0.0001**
Tumor grade				
I	Reference	Reference	Reference	Reference
II	**1.644**	**0.009**	1.217	0.327
III	**8.834**	**< 0.0001**	**2.633**	**< 0.0001**
IV	**34.977**	**< 0.0001**	**3.93**	**< 0.0001**
Race				
Asian	Reference	Reference		
Black	1.189	0.192		
Others	1.011	0.961		
White	1.091	0.437		

SD: standard deviation. Bold type is used to indicate that *p* is less than 0.05.

### 3.3. Correlation analysis

**Figure 2:**
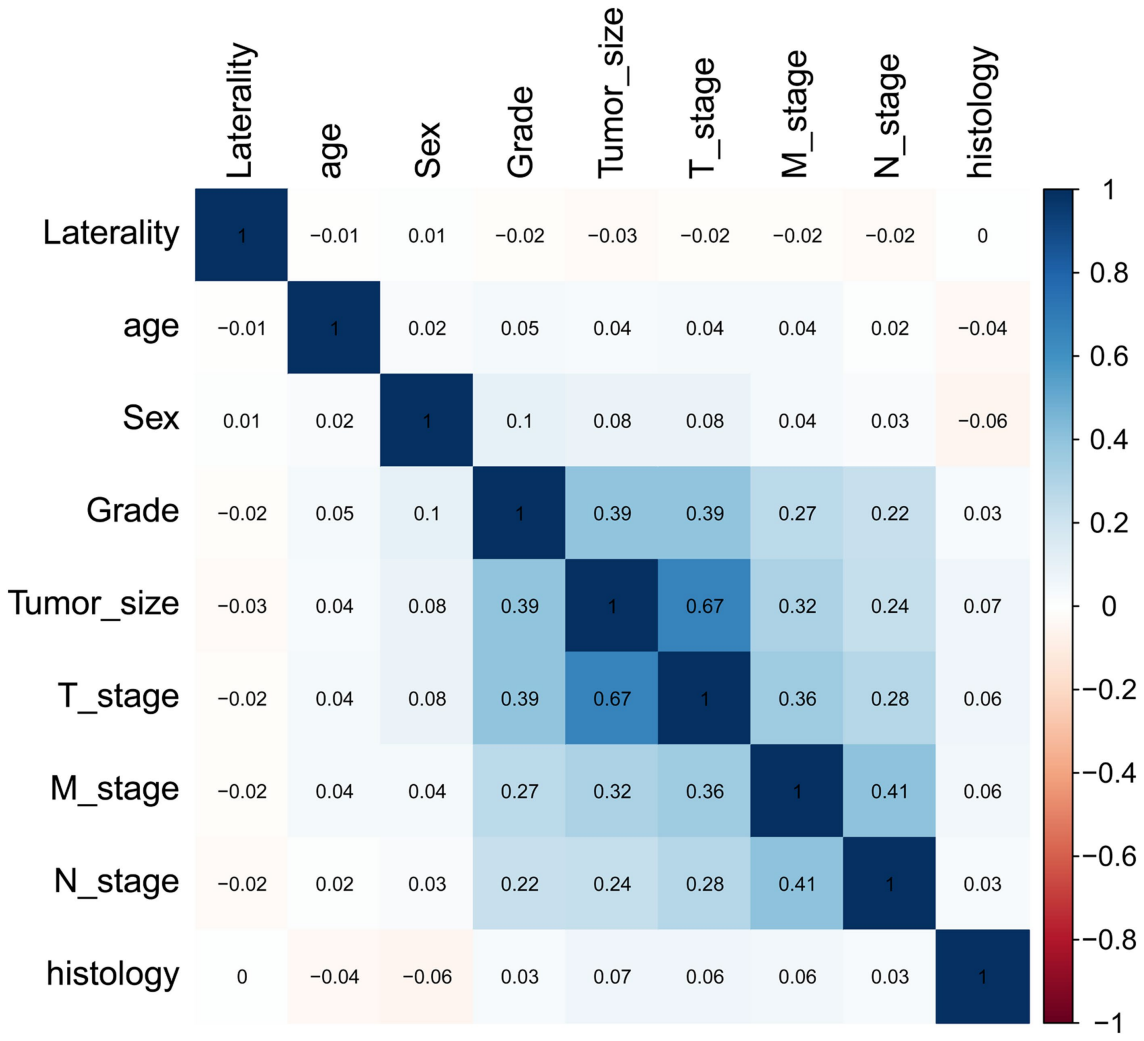
Heatmap of the correlation of patients’ clinical and pathological features.

The correlations between the variables selected as predictors were analyzed and visualized in a heatmap (Figure 2) using Spearman’s rank correlation coefficient. Among these, T stage, tumor size, tumor grade, and M stage were associated with N stage. However, none of the variables showed a strong linear relationship.

### 3.4. Performance of the machine learning algorithms

**Figure 3:**
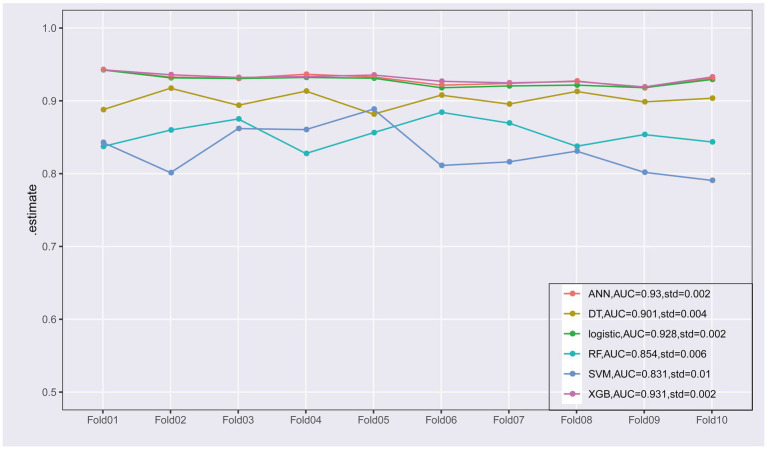
Ten-fold cross-validation of the receiver operating characteristic curves of the six machine learning models in the training cohort.

**Figure 4:**
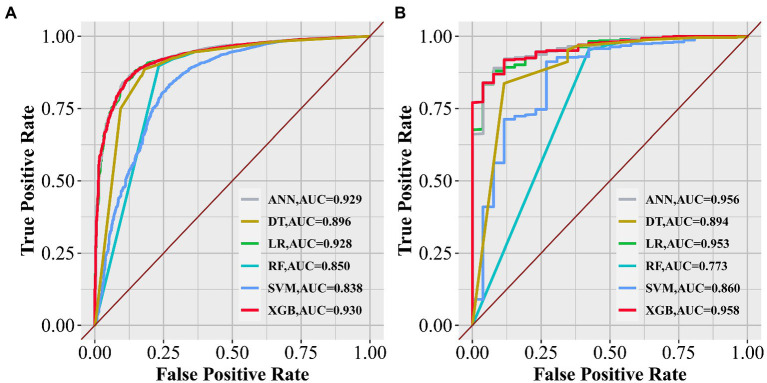
Receiver operating characteristic curves of the six algorithms in the internal **(A)** and external tests **(B)**.

**Figure 5:**
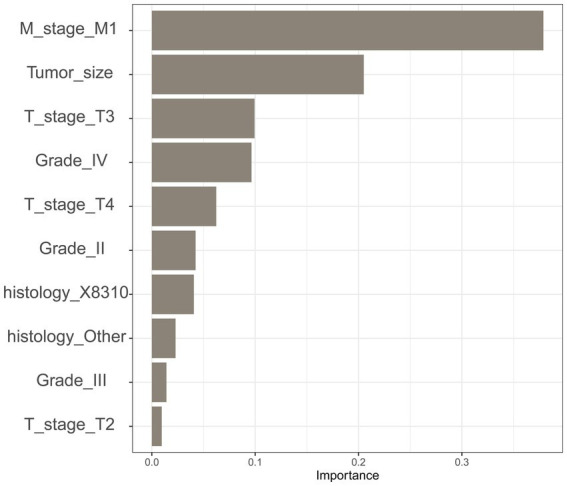
Feature importance ranks in extreme gradient boosting.

**Figure 6:**
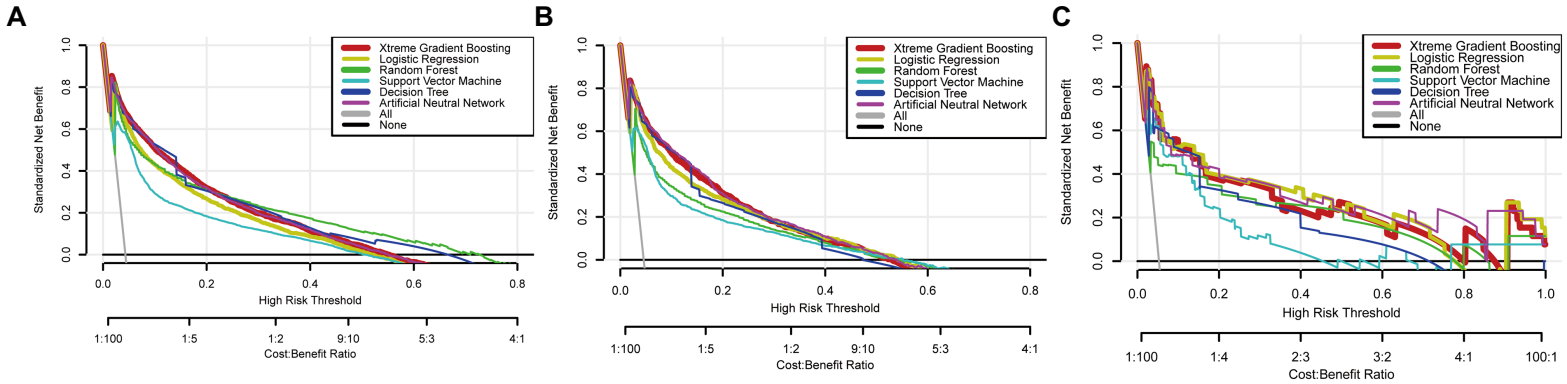
Decision clinical analysis curves of algorithms in the training **(A)**, internal test **(B)**, and external test **(C)** sets.

**Figure 7:**
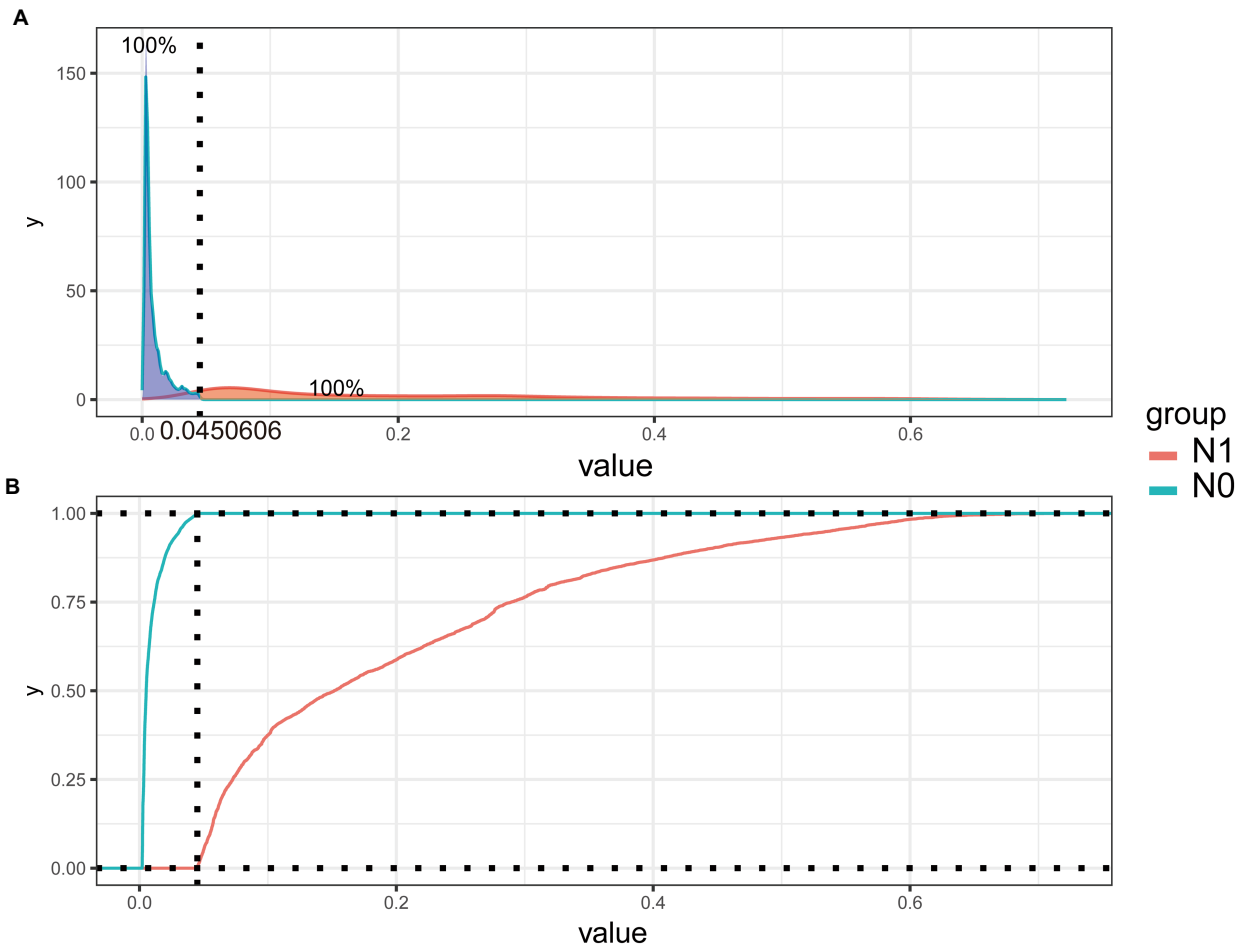
Probability density plot **(A)** and the clinical impact curve **(B)** of extreme gradient-boosting model.

To improve the stability and determine the optimal hyperparameters of the six ML algorithm models, a 10-fold cross-validation method was applied in this study. As shown in Figure 3, the XGB model had the best performance on ROC curve analysis (AUC = 0.931, Std = 0.002) of the six algorithms tested, and the ANN model also showed good performance (AUC = 0.930, Std = 0.002). The results of the comprehensive review process are shown in Table 4. XGB was superior to the other models in both internal and external trials. The AUC, accuracy, sensitivity, and specificity were 0.930, 0.857, 0.856, and 0.873, respectively, in the internal test and 0.958, 0.935, 0.769, and 0.944, respectively, in the external test. The ROC curves of the internal and external tests are shown in Figure 4. In the performance comparison of the ML algorithms, an AUC closer to 1 indicates that the model is better than models with lower AUCs. Therefore, we selected the XGB model as the final prediction model. We then ranked the importance of predictor variables for the XGB model using a permutation test (Figure 5). The M stage, tumor size, T stage, and tumor grade all had a marked impact on the forecast results. The DCA curve showed that XGB had the highest clinical applicability (Figure 6), which indicates that using the model could help clinicians to identify which patients with RCC may have LN metastases. The probability density plot (Figure 7A) depicting predictive distribution showed that the AUC was highest when the predictive score was 0.045. The CUC plot (Figure 7B) also showed good clinical applicability.

**Table 4:** Predictive performance of the algorithms’ internal and external tests.

Models		LR	RF	SVM	XGB	DT	ANN
Training	AUC	0.9	0.842	0.662	0.916	0.827	0.905
	Accuracy	0.788	0.903	0.947	0.813	0.953	0.793
	Sensitivity	0.876	0.747	0.388	0.881	0.079	0.874
	Specificity	0.783	0.911	0.976	0.81	0.998	0.789
Internal test	AUC	0.928	0.85	0.838	0.93	0.896	0.929
	Accuracy	0.85	0.891	0.849	0.857	0.882	0.842
	Sensitivity	0.881	0.768	0.695	0.856	0.82	0.887
	Specificity	0.848	0.897	0.857	0.873	0.885	0.84
External test	AUC	0.953	0.773	0.86	0.958	0.894	0.956
	Accuracy	0.933	0.933	0.935	0.935	0.937	0.921
	Sensitivity	0.731	0.577	0.538	0.769	0.654	0.885
	Specificity	0.944	0.953	0.957	0.944	0.953	0.923

ANN, artificial neural network; AUC, area under the receiver operating characteristic curve; DT, decision tree; LR, logistic regression; RF, random forest; SVM, support vector machine; XGB, extreme gradient boosting.

### 3.5. Web-based calculator

**Figure 8:**
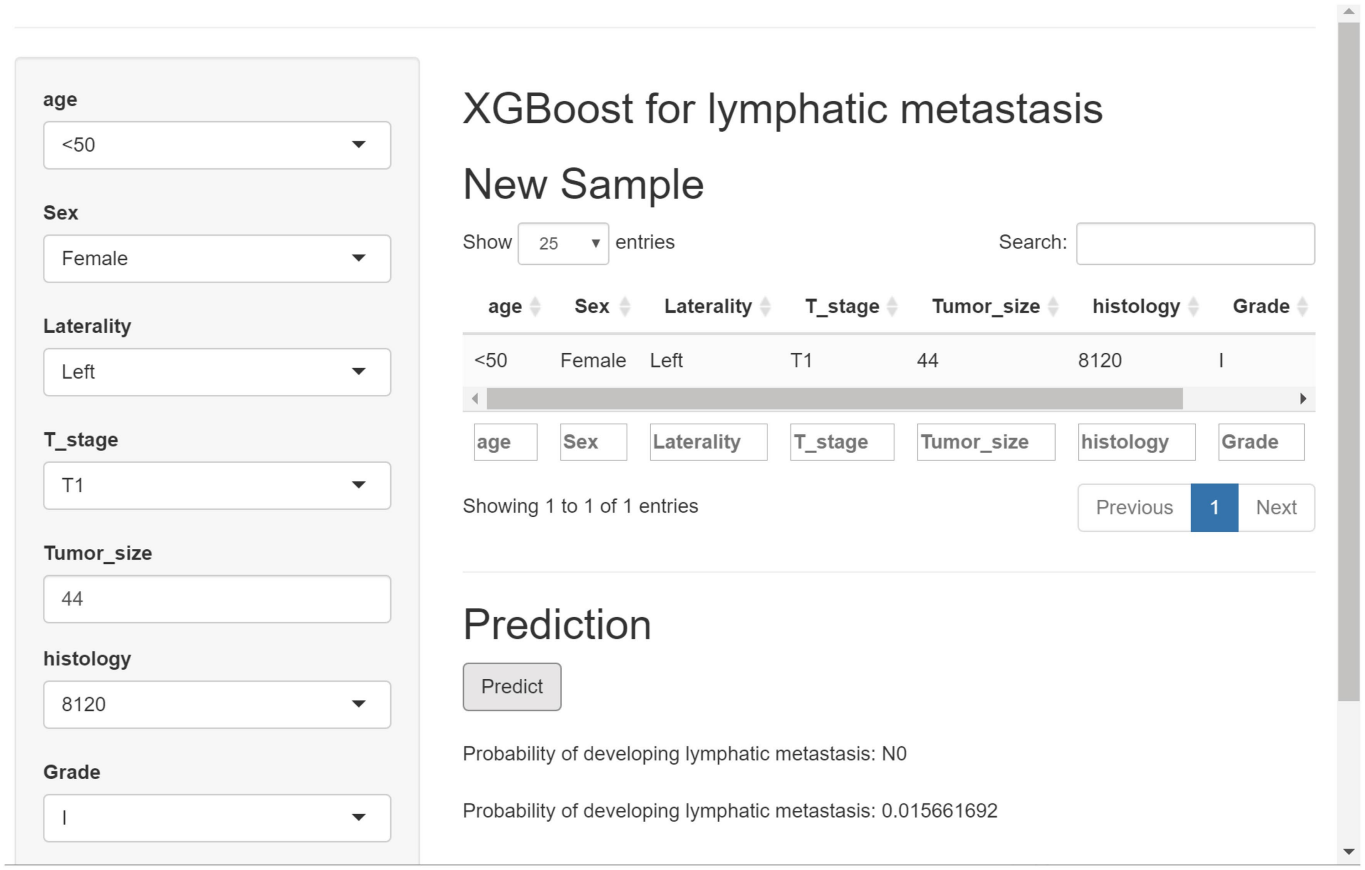
Web-based calculator created using the extreme gradient-boosting algorithm.

A web-based online predictor was built based on the most predictive XGB algorithm to enable clinicians to predict the risk of LN metastasis in patients with RCC.^1^ The calculator was easy to use, and physicians were able to enter variables in the option box to calculate the probability of developing LN metastasis for each patient with RCC (Figure 8). The result was automatically presented by clicking the “predict” button. The calculator was published online and can be found at: see text footnote 1.

### 3.6. Survival analysis

**Figure 9:**
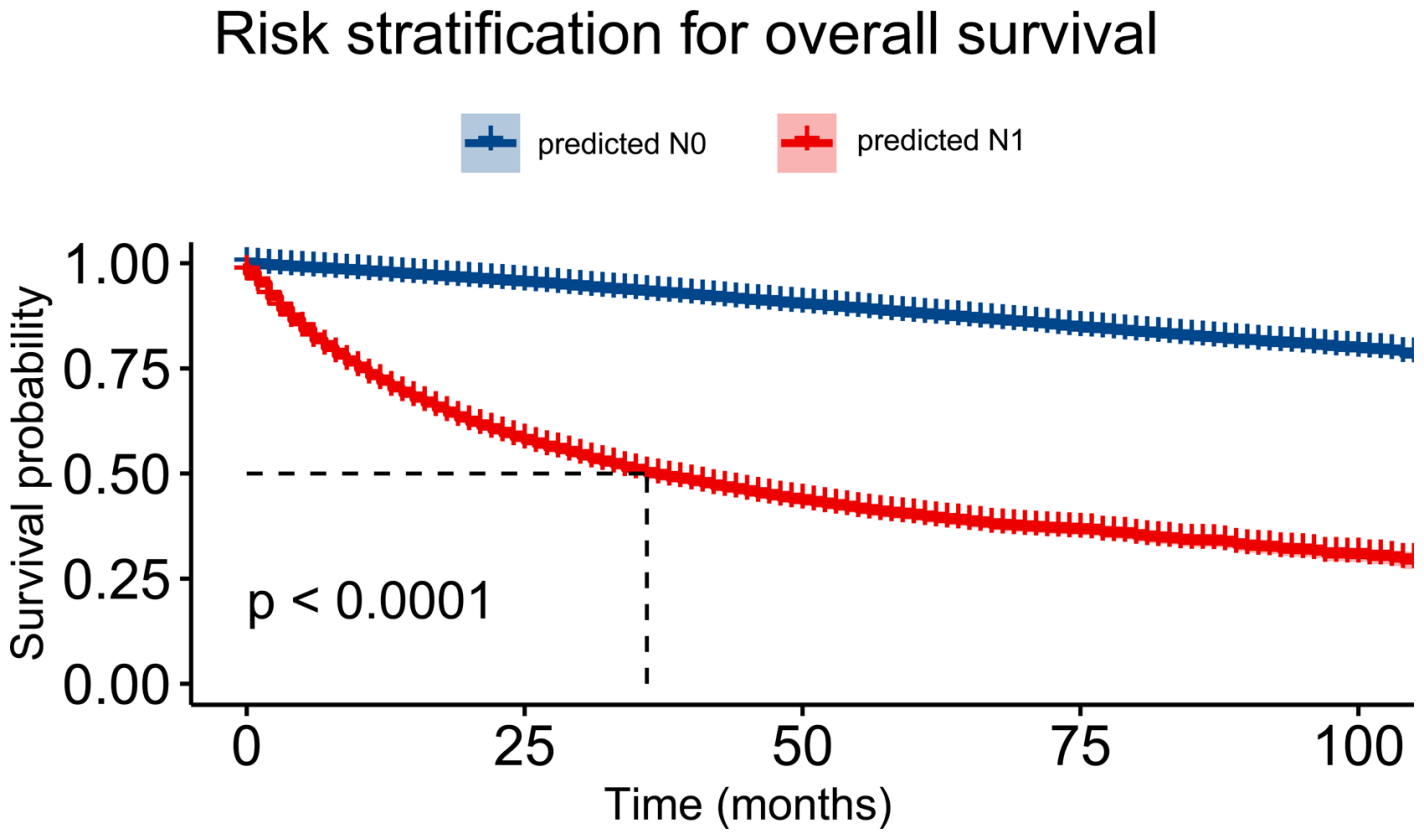
The Kaplan–Meier curve of overall survival comparing patients with N1 and N0 lymph node status based on the extreme gradient-boosting (XGB) model.

We analyzed the survival outcome according to the XGB predictive results. Survival analysis using the Kaplan–Meier method showed that the XGB model had fine discriminative ability for predicting OS (*p* < 0.0001; Figure 9). The survival analysis showed that patients with predicted N1 had a significantly shorter survival time (*p* < 0.0001).

## 4. Discussion

LN metastasis is the main metastatic pathway of RCC. Moreover, several studies have confirmed that patients with RCC with LN metastasis are more likely to develop distant metastasis (11). Once the lesion spreads to a distance, the 5-year survival rate decreases to ~10% (12). Surgical resection is still the primary treatment for RCC owing to its insensitivity to chemotherapy and radiotherapy. Recently, the value of LND in patients with RCC has been a topic of academic debate. Although some studies have not shown a benefit of LND, most studies suggest that LND promotes accurate tumor staging, improves the prognosis, and prolongs OS in patients with RCC (13–15). The 2018 European Association of Urology guidelines for RCC, state that patients with insidious involvement of lymph nodes (cN0/pN1 cM0) who undergo LND have improved prognosis and survival (16, 17). A clinical study revealed that some patients with RCC who underwent isolated LN resection, had good long-term survival (18). However, these recommendations are not supported by strong evidence, and previous studies have not identified which patients derive the greatest benefit from LND owing to the uncertainty of LN metastasis in renal cancer, which often make clinicians face difficulties in selecting the surgical method and scope.

Clinically, the diagnosis of LN metastases using CT or MRI is often difficult. Imaging examination has high specificity but relatively low sensitivity in the identification of LN metastasis in patients with RCC (19). The diameter of a normal LN is usually 1 cm as the upper limit of clinical imaging, but there are often undetected micrometastases (20). Therefore, a convenient tool to accurately predict LN metastasis is urgently required to guide LND. The resection of positive LNs is thought to be a direct therapy method that effectively blocks future LN metastasis.

Currently, precision medicine is summarized into four concepts: predictive, personalized, preventive, and participatory. Integrating big data with ML algorithms is becoming a clinical necessity. Recent large-scale studies have selected this novel and efficient means to investigate the clinical issues related to the early diagnosis and treatment of cancer metastasis; however, few studies have attempted to evaluate the risk of LN metastasis in patients with confirmed RCC using ML methods (21, 22). For example, Pin et al. (23) reported that patient age at surgery, largest tumor diameter, presence of preoperative symptoms, cT stage, cN stage, and serum biomarkers were associated with LN metastases in patients with RCC. Similarly, Wenle et al. (24) showed that a nomogram-based prediction method based on quantifying the risk of LN invasion in patients with RCC may have practical clinical application. Compared with traditional statistical methods, ML can construct an optimal mathematical model and constantly adjust the model parameters to effectively prevent overfitting. Therefore, in this study, six ML algorithms were developed and validated to predict LN metastasis in patients with RCC. Among them, the XGB model showed outstanding performance in both the internal and external validations, with an AUC of 0.930 and 0.958 in the internal and external tests, respectively. The DCA curve and CUC also showed good applicability.

Our analysis confirmed that the M stage, tumor size, T stage, and tumor grade were the four most important determinants in forecasting the results, which is consistent with the results of previous studies (10, 25, 26). Umberto et al. (10). found that the M stage at diagnosis and tumor size were the most important independent predictors of LN metastases. In addition, patients with RCC with a tumor diameter > 7 cm (cT2a or higher) have been shown to have a significantly increased risk of LN progression (25). Several researchers have found that LN metastases in patients with RCC are dependent on many factors, but especially on tumor size and tumor grade. Pantuck et al. (26) found that only 6% of tumors with Fuhrman grades 1–2 had LN involvement, and 26% of tumors with high Fuhrman grade had LN involvement. In patients with RCC, LN metastasis is more common in men than in women. Smoking and alcohol consumption are well-established risk factors in RCC (27) and are associated with a relatively high incidence of LN metastasis in men with RCC. A novel finding of this study is that patients with renal cancer whose tumor site was on the left side had a higher risk of LN metastases. A retrospective study using two different national databases showed that patients with left-sided RCC were more likely to have a higher tumor grade, LN positivity, and distant metastasis than those with right-sided disease (28). The spread patterns of the lymphatic system may have contributed to these differences. The effect of the side of the tumor on prognosis warrants further in-depth study.

This study has several strengths. Based on a large sample size and internal and external validations, our study ensured credibility and authenticity. In addition, the survival outcomes of the N1 group were worse than those of the N0 group, which is consistent with the results of previous studies. Several studies have shown that 5-year survival rates are often <40% for patients with LN involvement (18, 29). This further indirectly verified the reliability of the prediction results of this study. Nevertheless, this study also has some limitations. First, the use of retrospective data may have led to data bias. Second, this study did not include specific biochemical indicators. Although this avoids the effects of differences in levels of testing at different institutions, some specific biochemical parameters that were omitted warrant further investigation. In addition, owing to the inevitable differences in the level of diagnosis and treatment in different countries or regions, the external validation cohort only included Chinese patients, and so the generalizability to other countries is unclear. However, our study represents an important step forward in developing a model to predict the risk of LN metastasis in patients with confirmed RCC.

In conclusion, our study showed that integrating ML algorithms and clinical data can effectively predict LN metastasis in patients with RCC. Subsequently, a freely available online calculator (see text footnote 1) based on the XGB model was built to quantify the risk of LN metastasis in patients with RCC conveniently and accurately.

## Data availability statement

The raw data supporting the conclusions of this article will be made available by the authors, without undue reservation.

## Ethics statement

The studies involving human participants were reviewed and approved by Ethics Committee of the First Affiliated Hospital of PLA Army Medical University. The patients/participants provided their written informed consent to participate in this study.

## Author contributions

YZ and XY: conceptualization. KP: methodology and project administration. XY: software. YZ: validation, writing—original draft preparation, and writing—review and editing. PX: formal analysis. ZT: investigation. NY: resources. LZ: data curation. QD: visualization. HL: supervision. All authors contributed to the article and approved the submitted version.

## Funding

The work was funded by the National Natural Science Foundation of China (authorization number: 81873606).

## Conflict of interest

The authors declare that the research was conducted in the absence of any commercial or financial relationships that could be construed as a potential conflict of interest.

## Publisher’s note

All claims expressed in this article are solely those of the authors and do not necessarily represent those of their affiliated organizations, or those of the publisher, the editors and the reviewers. Any product that may be evaluated in this article, or claim that may be made by its manufacturer, is not guaranteed or endorsed by the publisher.

## Footnotes

^1^ https://xinglinyi.shinyapps.io/20221004-app/
